# Effect of femtosecond laser interaction with human fibroblasts: a preliminary study

**DOI:** 10.1007/s10103-023-03740-2

**Published:** 2023-03-03

**Authors:** M. A. Zaki Ewiss, M. A. Mahmoud, R. Steiner

**Affiliations:** 1 Department of Physics, Faculty of Science, Cairo University, Giza, 12630, Egypt; 2 Department of Pathology, Faculty of Veterinary Medicine, Cairo University, Giza, 12211, Egypt; 3 Institute of Laser Technologies in Medicine and Metrology at the University of Ulm, 89081 Ulm, Germany

**Keywords:** Femtosecond laser, Autofluorescence, Fibroblast, Co-enzymes, Cell culture

## Abstract

In in vitro methods and cell culture models, femtosecond (fs) laser interaction has been employed to assess its effect on the proliferation and morphology of human skin fibroblasts. We cultured a primary human skin fibroblast cell line on a glass plate, passages 17–23. The cells were irradiated with a 90-fs laser at a wavelength of 800 nm and a repetition rate of 82 MHz. The target received an average power of 320 mW for 5, 20, and 100 s, corresponding to the radiation exposures of 22.6, 90.6, and 452.9 J/cm^2^, respectively. Using a laser scanning microscopy technique, the photon densities were measured to be 6.4 × 10^18^, 2.6 × 10^19^, and 1.3 × 10^20^ photons/cm^2^ in a spot area of 0.07 cm^2^; the recorded spectra were obtained from the laser interaction after 0.00, 1.00, 25.00, and 45.00 h. The cell count and morphological changes showed that the cultured cells were affected by laser irradiation under photon stress; some fibroblasts were killed, while others were injured and survived. We discovered evidence of the formation of several coenzyme compounds, such as flavin (500–600 nm), lipopigments (600–750 nm), and porphyrin (500–700 nm). This study is motivated by the future development of a novel, ultra-short fs laser system and the need to develop a basic in vitro understanding of photon–human cell interaction. The cell proliferation indicated that cells are partly killed or wounded. The exposure of fibroblasts to fs laser fluence up to 450 J/cm^2^ accelerates cell growth of the viable residual cell.

**Supplementary Information:**

The online version contains supplementary material available at 10.1007/s10103-023-03740-2.

## Introduction

The main cells that make up the connective tissue are fibroblasts, found in human organs and tissues containing extracellular matrix (ECM) components. In 2001, Ivanovski et al. stated that the essential functions of fibroblasts are the synthesis and homeostatic balance of the ECM in tissues and organs [1]. Fibroblasts are highly metabolically active cells that express and secrete most ECM components (collagen, proteoglycan, fibronectin, tenascin, and laminin) [2]. Metabolically active cells are critical in regulating the ECM, intracellular fluid volume, and wound healing pressure. Fibroblasts can also be transformed into other cells, particularly osteocytes [2, 3].

Several studies, either in vivo and/or in vitro, have focused on implementing native fluorescence or autofluorescence (AF) in medical applications in the ultraviolet (UV)–visible and near-infrared (IR) spectral range when medical or biological substrates are excited with light at a suitable wavelength [4–10]. The strict relationship between several endogenous fluorophores and living systems’ morphofunctional properties influences the AF emission features.

Medical therapies for various illnesses include low-level laser therapy (LLLT), also known as low-intensity light therapy (LILT), cool laser, phototherapy, light therapy, low-energy laser therapy, and photobiomodulation.

A single wavelength of light is produced by the non-invasive light source treatment known as LLLT. It makes no vibrations, noise, or heat. It is also known as biostimulation or photobiology. In this method, lasers create a narrow, concentrated, monochromatic beam of electromagnetic energy (one wavelength) that concentrates the wavelengths in a specific area. Depending on the type of laser, the wavelength range of electromagnetic radiation encompasses the visible light spectrum and infrared light [11].

Several studies have reported more complex responses of fibroblasts at different energy densities or wavelengths of LLLT. Kreisler et al. [12] used the fluorescence activity of the REDOX indicator to assess the effect of GaAlAs diode laser (809 nm, 1.96–7.84 J/cm^2^, 1–3 treatments) on the proliferation of human periodontal ligament fibroblasts. They found a significant increase in cell proliferation up to 72 h after LLLT. Schertinger et al. [13] reported similar results in the MTT (3-(4,5-dimethylthiazol-2-yl)-2,5-diphenyltetrazolium bromide) assay (0.37 ± 0.11 vs 0.23 ± 0.10, *p* < 0.001). On the other hand, Hawkins and Abrahamse [14] investigated the response of injured human dermal fibroblasts to HeNe (632.8 nm) laser treatment at various doses of 2.5, 5.0, and 16.0 J/cm^2^, 1–3 times daily for two consecutive days. Their results showed that 2.5 J/cm^2^ for 2–3 daily treatments and 5.0 J/cm^2^ for once daily treatments increased cell proliferation and migration while maintaining cell viability without stress or injury. Periodontal ligament stem cells and gingival fibroblasts’ responses to 980-nm diode laser irradiation have both been investigated [15]. In this experiment, cells were irradiated with energy densities of 0.5, 1.5, and 2.5 J/cm^2^ using a continuous-wave 980-nm diode laser with a 200-mW output. The results showed that the laser radiation positively stimulated the viability of these cells. Another study also showed that irradiation with a Ga-As diode laser (904 nm) at 3 or 4 J/cm^2^ for 1–6 days increased the number of NIH-3T3 fibroblasts by 3- to 6-fold compared to controls. However, high energy densities of 5 J/cm^2^ did not stimulate cell proliferation [16]. Similar results have been found in other studies [17] to confirm the biostimulatory effects of LLLT in a limited range of energy densities, and excessively high energy densities may lead to the opposite impacts.

Besides the energy density, this investigation also focused on the effects of different wavelengths on fibroblast proliferation. Crisan et al. [18] compared the impact of 830, 980, and 2,940 nm lasers (5.5 J/cm^2^) on human skin fibroblasts by MTT and apoptosis assays. They showed that both 830 nm and 980 nm significantly stimulated cell proliferation at 24, 48, and 72 h after irradiation, whereas 2940 nm inhibited cell proliferation and promoted apoptosis. In 2018, Ma et al. conducted LLLT in vitro studies on human fibroblasts’ proliferation and collagen synthesis [5]. They used a continuous wave (CW) diode laser to irradiate healthy human fibroblasts at wavelengths 635, 800, and 635 + 800 nm with the same energy density of 60 J/cm^2^. The healthy cells showed proliferation and collagen synthesis when irradiated at 635 + 800 or 800 nm.

It is worth mentioning that most studies on the development of LLLT do not record the duration between the laser exposure and the evaluation. It has been established that a single dose of 5 J/cm.^2^ stimulates cell proliferation and mitochondrial activity, which normalizes cell function and accelerates wound healing [8]

The development of laser technology provided access to lasers that could provide focal spots with higher energy density. Nowadays, ultrafast lasers known as a femtosecond (fs) lasers emit light pulses with a duration much below one picosecond or in the region of femtoseconds. One femtosecond equals 10^−15^ s. Mode interlock produces these impulses. The femtosecond oscillator’s output can be used directly for medical applications or amplified to greater impulse energies. Applications requiring either very high time resolution or very high peak intensity can use fs lasers. The energy in each pulse is also confined in a very short timescale due to the short pulse duration and low duty cycle, resulting in exceptionally high peak power. In 2017, Heitz et al. implemented the fs laser–induced microstructures on Ti substrates to minimize cell adhesion [6]. They showed that fs laser irradiation of metallic Ti substrate at a wavelength of 790 nm resulted in sharp conical spikes, forming a periodic surface structure. This structure creates a cell–hydrophilic repellent surface. In addition, they discovered that these surfaces could be applied in medical implants or prostheses. The combined effect of the fs laser treatment and anodization on the microstructure and fibroblast cell growth properties of Ti6A14V alloy have been recently reported [7].

The mechanism of laser beam interaction at the cellular and tissue level has been studied [9]. The aim was to understand the operation and effectiveness of the laser beam effect at the cellular and tissue levels. Various laser sources and the dosimetry principles of laser therapy applications have been reviewed [9]. Fs laser applications for biopreservation and profound implications for surgery and cell isolation have been investigated. The non-invasive manipulation of live cells plays an essential role in cell-based therapeutics. Also, the fs laser pulses were used to study the cellular manipulation and the generation of optical pores for cytoplasmic delivery of nonreducing cryo-protectants. Laser therapy induces increased viability and proliferation in isolated fibroblasts [10]. Their study evaluated how laser therapy could improve wound healing in fibroblasts in vitro. Notably, fibroblast cell proliferation and mitotic activity increased as the light intensity increased, consequently increasing the likelihood of wound healing.

High-intensity fs lasers can disrupt nanoscale structures, such as intracellular organelles. They modify biological functions reversibly, known as nanosurgery biophoto modulation [19]. Moreover, surface-ablation resolution at nanometer-scale precision has been investigated [20]. The optical damage caused by fs laser pulses and their applications to nanomachining has been determined [21]. The fs laser cell interaction, nonlinear processes, and optical breakdown have been investigated [22]. Significant macular regeneration has been observed by exposing the macula to nanosecond laser pulses [23]. Age-related macular degeneration containing macular deposits has been ablated using laser pulses between 10 and 106 fs at a wavelength range of 200 nm–30 µm [24, 25]. During depth targeting, extracellular macular deposits can be removed, depleted, denatured, and destroyed without damaging the retinal pigmented epithelium cell membrane or macular [26]. The ultra-short pulsed laser technique is highly localized, and the nanosurgical procedure is contained entirely within the focal volume of the focused femtosecond beam. Adjacent material is undisturbed, and no cell collapse or morphology is seen. Interestingly, disruption of focal adhesions detaches the fibroblast cell from the adjacent cell, and the cell responds by folding, thereby isolating the single mammalian cell post-nanosurgery. A focused fs laser pulse technique has been proposed to create embryonic manipulation, biopreservation, drug delivery, and gene therapy.

In addition, atherosclerotic plaque ablation using ultrafast laser pulses for CVD has been reported [26]. Ultrafast lasers generate extremely short light pulses, mainly of the order of picoseconds or femtoseconds. These lasers depend on techniques like mode-locking to form a train of pulses. Furthermore, fs laser irradiation produces excess intracellular reactive oxygen species [27]. Thus, they induced apoptosis-like cell death in a mitochondria-dependent manner in irradiated cells without damaging adjacent cells in primary cultured smooth muscle cells. External forces such as a short-pulse electric field or laser radiation could stress living cells. Biological studies of human fibroblasts’ cellular constitution have been previously reported [28–32]. Under certain circumstances, the stressed living cells could produce other chemical compounds to protect or repair the damaged DNA.

We hypothesized to determine whether fs laser irradiation of fibroblasts could produce AF and if this phenomenon is due to enzymatic activation. This study investigates the effect of fs laser irradiation on fibroblast proliferation and morphology. The excitation band and corresponding AF pattern demonstrate the formation of molecules. Natural fluorophores of flavins, porphyrins, and lipopigment coenzymes were investigated at various fs laser radiant exposures.

## Experimental arrangements

### The cell culture procedures

A primary human-skin fibroblast cell line, passages 17–23, was used in this experiment [33]. The cell culture was prepared using Invitrogen (41966–029) and adding 1% penicillin/streptomycin, 1% L-glutamine, and 10% fetal calf serum (Biochrom A2213, K0282, and S0115), respectively. A 100-ml solution of Trypsin EDTA 10 × (Invitrogen 15400–054) was diluted with PBS (1:10). A new cell culture vessel (e.g., T-25 flask) was filled with the respective cell culture medium (less than Trypsin EDTA 10 ×). The frequency of refreshing the medium depends on the cell line’s growth factor. The fibroblasts were placed in a glass Petri dish for further experimental investigation.

### Femtosecond laser irradiation

**Fig. 1:**
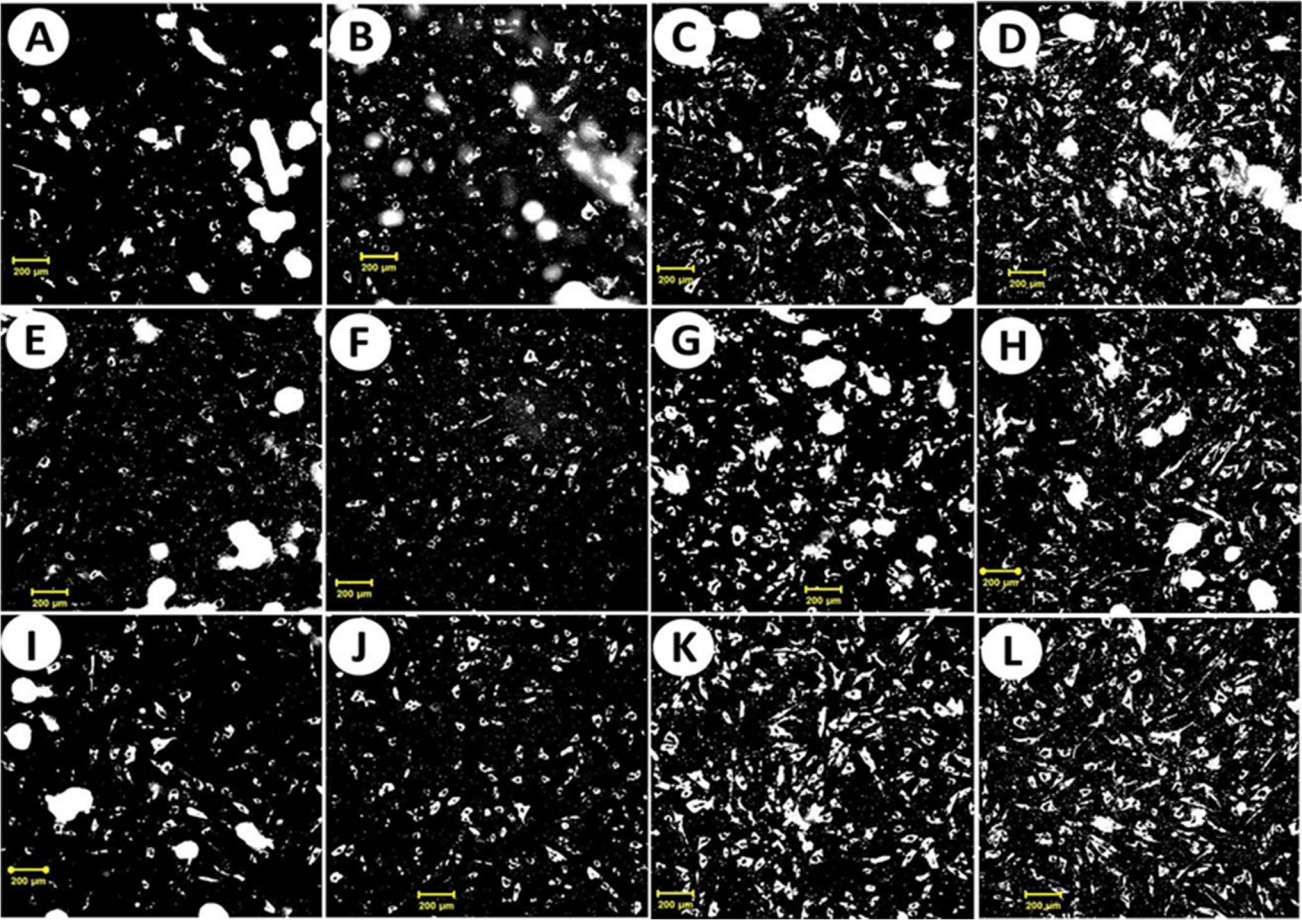
Cell count image of fibroblast on glass plate irradiated with 90 fs laser at a wavelength of 800 nm with a repetition rate of 82 MHz and an average power at the cell target of 320 mW, with exposure times of 5 s, 20 s, and 100 s after incubation at 0:00 h (**A**, **E**, **I**), 1:00 h (**B**, **F**, **J**), 25:00 h (**C**, **G**, **K**), and 45:00 h (**D**, **H**, **L**) from the laser irradiation, respectively (scale bar 200 µm)

The fibroblasts were irradiated with fs laser pulses generated by a Spectra-Physics fs laser system. This system comprises two main components: the Spectra-Physics, Millennia Vs serial number 568 model MILLVsS [34], and Tsunami, serial number 1953 model 3960-M3S (Spectra-Physics Tsunami Operation Manual). Table 1 and Fig. 1s show the laser output characteristics and the laser irradiation experiment’s optical configuration. Millennia Vs is a CW Nd:YVO laser. The main wavelength of 1064 nm is an intracavity frequency doubling by an LBO crystal to output a wavelength of 532 nm. The average power output to pump the Tsunami is 5 W.

**Table 1:** Laser output characteristics

Millennia Pros Laser Model MillVsS		Tsunami model 3960-M3S	
Output characteristics		Output characteristics	Ultra-short pulse (5 W pump)
Power	5 W	Tuning range	780–850 nm
Wavelength	532 nm	Average power	650 mW at 800 nm
Spatial mode	TEM_00_	Pulse width	90 fs
Beam ellipticity	< 10%	Peak power	> 125 kW at 800 nm
Beam diameter at 1/e2 points	2.3 mm ± 10%	Pulse energy	~ 6 nJ
Beam divergence, full angle	< 0.5 mrad ± 10%	General tsunami specifications	
Polarization	> 100:1 vertical	Repetition rate (nominal)^1^	82 MHz
Power stability	± 1%	Noise	< 0.2
Beam pointing stability	≤ 2 µrad/°C	Stability	< 5%
Noise	< 0.04% rms	Spatial mode	TEM_00_
Boresight tolerance		Beam diameter (1/e^2^)	< 2 mm
Near field	± 0.25 mm	Beam divergence, full angle	< 1 mrad
Far field	< 3 mrad	Polarization	> 500:1 vertical

The Tsunami is an active mode-locked fs oscillator. The emitted laser beam’s wavelength can be selected from 720 to 850 nm. The pulse duration was 90 fs with a repetition rate of 82 MHz, which depends on the cavity’s geometry. The average output power of our Tsunami was 650 mW. The laser beam was deflected using mirrors to reach the target. There was a loss of power at each mirror (the average power of the laser beam that reached the target was 320 mW from the Tsunami (82 MHz)). The pulse duration at full-width half maximum (FWHM) and the Tsunami wavelength were measured using an autocorrelator and a spectrometer; both values were monitored using a Pulse Scope (APE, Berlin, SerNo. 00/4A33).

The Tsunami fs laser’s emission wavelength was 800 nm in all experiments. The laser beam was deflected by mirrors and focused using a biconvex lens (*f* = 150 mm). The glass Petri dish is placed in front of the laser beam (at off focus point) with a spot area of 0.07 cm^2^. Four circles with different colors (with an area of 0.07 cm^2^) were labeled in a glass Petri dish. One circle was considered for unirradiated fibroblasts and was used as a control sample. Thus, the region of interest of 2 × 2 mm^2^ was fully illuminated; this point was before the lens’s focal length and thus before the laser beam’s focus (Fig. 1). The laser exposure time was 5, 20, and 100 s, corresponding to total laser radiant exposures of 22,6, 90.6, and 452.9 J/cm^2^, respectively. The photon densities that struck the cells were 6.4 × 10^18^, 2.6 × 10^19^, and 1.3 × 10^20^ photons/cm^2^.

### Laser scanning microscope

The spectral characteristics were investigated using a laser scanning microscope [35]. The 488-nm laser line of the 30-mW Argon laser was used for excitation with 50% of its maximum power. The detector’s resolution was set to 10.7 nm per channel in a 503- to 717-nm bandwidth. The 2.5/0.075 Plan-Neofluar, 10 × /0.3 Plan-Neofluar, and LD 40 × /0.6 corrected objectives were used. A pixel time of 6.4 µs was obtained when capturing the spectra with a 512 × 512 pixel resolution over a scanning field with a square size of 921.4 µm. The pinhole was close to 169 µm, equivalent to 3.41 Airy Units (1 Airy Unit = 0.61 × emitted wavelength × total magnification / number of apertures (NA)). To minimize noise, we set an amplifier to an offset of 0.072 V with a detector gain of 869 V. Four scan lines should be averaged to achieve an acceptable signal-to-noise ratio.

### Cell count and proliferation rate measurement

The cell count of different groups was performed using the software (ImageJ, 1.x, NIH, USA).

## Results and discussion

### Cell count and proliferation rate

**Fig. 2:**
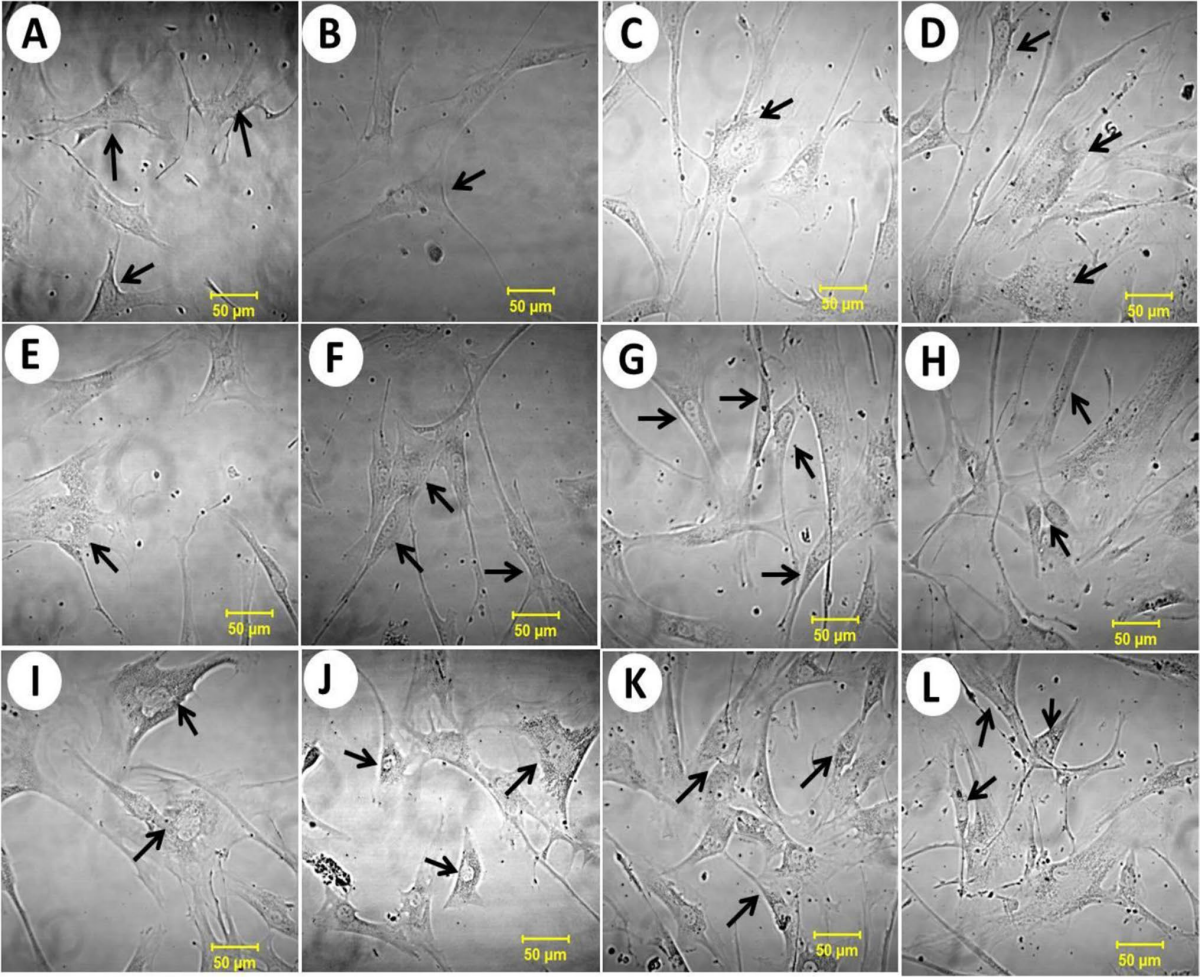
Morphology of fibroblast on glass plate irradiated with 90 fs laser at a wavelength of 800 nm with a repetition rate of 82 MHz and an average power of 320 mW at the cell target, with exposure times of 5 s, 20 s, and 100 s after incubation at 0:00 h (**A**, **E**, **I**), 1:00 h (**B**, **F**, **J**), 25:00 h (**C**, **G**, **K**), and 45:00 h (**D**, **H**, **L**) from the laser irradiation, respectively (scale bar 50 µm)

The unirradiated fibroblasts were considered a control sample; the normal fibroblasts were spindle-shaped and slender. Figure 2s and Table 2 show the unirradiated fibroblast cell count after 0.00, 1:00, 25:00, and 45:00 h incubation time. The results of the cell population of normal unirradiated cells were compared with those of the population after irradiation at different exposures and incubation times. The unirradiated cells initially proliferated at a rate of 7.69%. This value is significantly lower than the 141.07% obtained after 25 h incubation. After that, the proliferation slightly decreased by 14.8% after 45:00 h of incubation. This observation was consistent with [36, 37], who described this effect as the result of numerous cell-ageing mechanisms.

**Table 2:** Fibroblast cell density cultured in glass plate irradiated with 90 fs laser beam at a wavelength of 800 nm with a repetition rate of 82 MHz and an average power of 320 mW at the cell target at different exposure times

Incubation time (h)	Cell counts Fibroblast on a glass plate							
	Laser exposure time (s) (energy density (J/cm^2^))						Unirradiated cells	
	5 s (22.6 J/cm^2^)		20 s (90.6 J/cm^2^)		100 s (452.9 J/cm^2^)		Cell count	Proliferation rate (%)
	Cell count	Proliferation rate (%)	Cell count	Proliferation rate (%)	Cell count	Proliferation rate (%)		
0:00	49	+ 0.00	38	0.00	88	0.00	52	0.00
1.00	103	+ 110.02	89	+ 134.21	130	+ 47.73	56	+ 7.69
25.00	138	+ 33.98	103	+ 15.73	177	+ 36.16	135	+ 141.07
45.00	149	+ 7.97	99	− 3.88	179	+ 1.13	115	− 14.8

Alternatively, cellular proliferation was affected by the total laser radiant exposures of 22.6, 90.6, and 452.9 J/cm^2^ (Fig. 1; Table 2). Some cells were recovered after 1:00 h incubation and their proliferation rate increased. Our findings agreed with Cuerda-Galindo et al. [38], who discussed the effect the pulsed light 800–1200 nm emitting near-infrared in vitro cultured fibroblasts cells; in their report, evidence indicates that increases in fibroblasts proliferation and activity together with increases in some extracellular matrix proteins.

Our results showed that by increasing radiation exposure, some cells were killed while others were damaged.

Consequently, the incubation period after 25:00 and 45:00 h following laser irradiation impacted the proliferation rate. Notably, the proliferation rate increased slightly in all cases after 25:00 h incubation. These values were compared with the dramatically increased proliferation rate of 141% for unirradiated cells. The cell count decreased in all cases after 45:00 h of incubation, including the unirradiated cells. The irradiated cells recovered by generating repairing coenzymes. This effect may be attributed to the cell–photon stimulation, which depends on the photon density, exposure time and experimental conditions [39, 40].

### Cell morphology

**Fig. 3:**
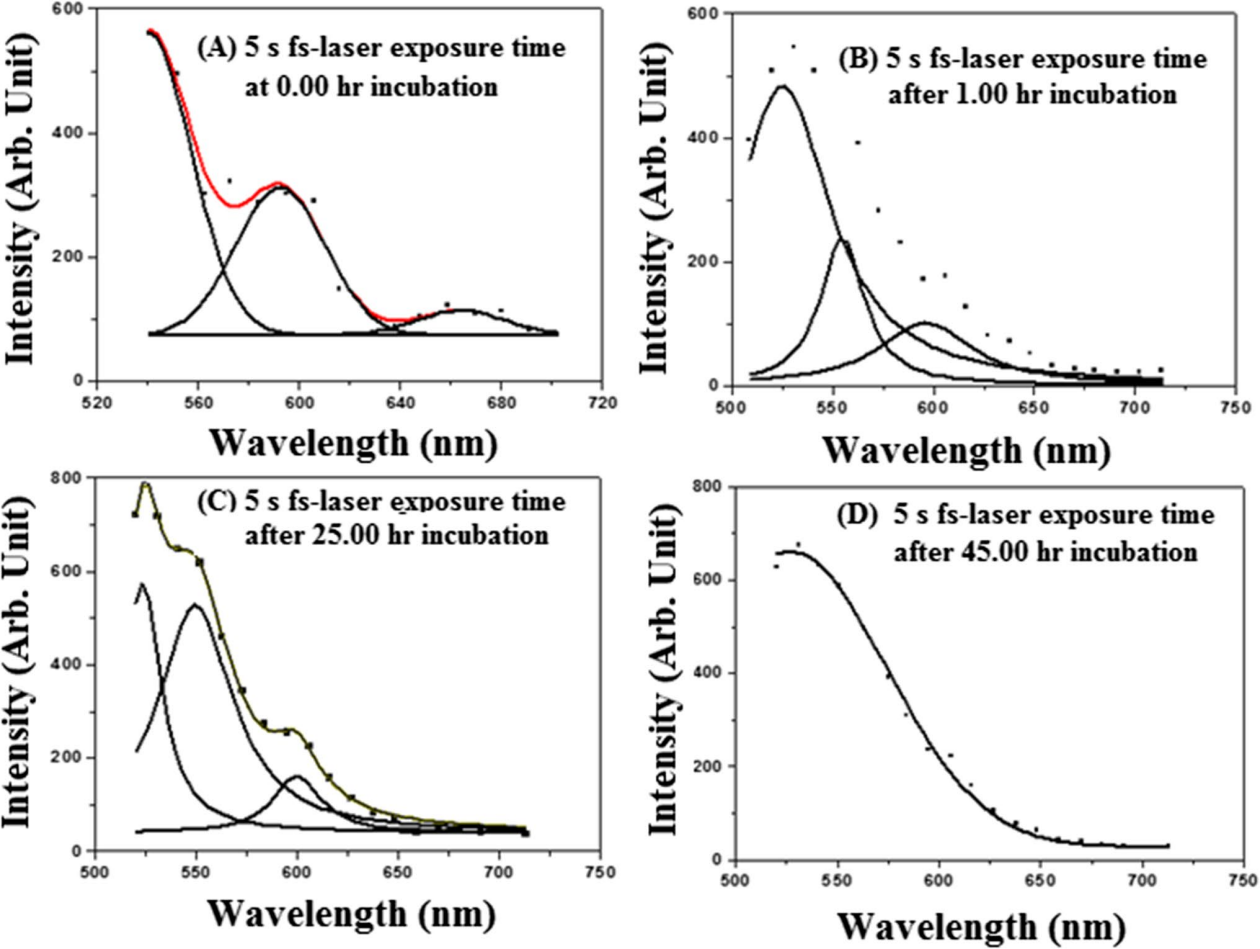
De-convoluted autofluorescence spectra of the fibroblasts irradiated for 5 s with 90 fs laser beam at 800 nm wavelength and an average power of 320 mW. (The red line indicates the fitting curve of the experimental data.)

The cells’ morphological changes were described using the proposed model [41, 42]. Figure 3s shows the images of normal cell morphology before irradiation. The most common morphological changes showed that the irradiated groups are affected to various degrees (Fig. 2). The cell group irradiated with 65 J laser energy for 100 s experienced the most harmful effect. The cells in this group displayed cytoplasmic lysis and lost details of the nuclei. Other groups showed varying degrees of slight cellular changes, including cellular adhesion, losing both cytoplasmic granules and cell processes. The morphological alterations of human fibroblasts exposed to various forms of electromagnetic radiation have been discussed in the literature [43]. It is worth mentioning that the morphological aspect and cells’ organization, either in the cell line or in the tissues, are influenced by the normal functions and integrity of the cell wall. So, the damage to the cells is expected to have a marked effect on cellular morphology. In this regard, the impact of cellular damage resulting from the irradiation of human skin fibroblast in cell lines or in vivo applications for oncological studies has been discussed in [44, 45].

Using different laser parameters in various treatment studies makes it more challenging to make meaningful comparisons. The therapeutic photobiological effect, for instance, is linked to the non-thermal photochemical or photobiological action of light through contact with a variety of endogenous photoreceptors and chromophores present in human fibroblasts [39]. The LLLT, on the other hand, uses low radiation intensities and has an output of up to 500 mW; it has been shown to have stimulatory, anti-inflammatory, and analgesic effects. According to the characteristics of the light itself, such as wavelength and coherence, this kind of laser can change intercellular communication, which in turn alters cellular processes. Additionally, laser light influences the mitochondrial respiratory chain by enhancing the activity of specific enzymes and accelerating the formation of collagen and pro-collagen [46]. The results of the laboratory studies on the cellular mechanisms of low-level laser therapy showed an increase in proliferation and supported the clinical outcome of improved wound healing. Another important observation from the laboratory studies is the inverse relationship between the power or energy density of LLLT and the cellular response. Comparative analyses showed reduced effects of higher energy densities, and paradoxically, further increases resulted in inhibition of cell proliferation, migration, viability, or ATP activity. Although LLLT promoted fibroblast cell differentiation, the response did not appear to be sensitive or inversely related to higher energy densities, as is the case for cell proliferation [47].

Meanwhile, there is limited information on how fs laser irradiation affects wound healing; however, LLLT affects the two main factors of wound healing—fibroblast cell proliferation and collagen synthesis [5]. Therefore, complementary studies on the potential impact of fs laser irradiation on wound healing are expected because our results included fibroblast cell proliferation, which is another essential factor that may enhance wound healing. However, the type and time of radiation exposure significantly impact the morphological changes in irradiated fibroblasts. Regarding this issue, Ma et al. [5] described some morphological changes in human fibroblasts exposed to low-level laser therapy and its effect on collagen synthesis.

In Fig. 2, we show the changes in the direction of cell growth and morphology of the irradiated cells with laser exposure times of 5, 20, and 100 s after incubation at 0:00 h (A, E, I), 1:00 h (B, F, J), 25:00 h (C, G, K), and 45:00 h (D, H, L), respectively. The fibroblasts exposed to high radiation exposure were more affected than the other cells; this result may be attributed to the frustration caused by photon stress [44].

At this point, our work needs to be expanded to account for various fs laser wavelengths, pulse durations, and peak powers to fully understand how fs laser irradiation impacts wound healing.

### Autofluorescence spectra

The fluorophore classes—endogenous, endogenously synthesized, and exogenous—each has distinct advantages and limitations for various clinical applications. Endogenous fluorophores are associated with the structural matrix involved in cellular metabolic processes [4]. The most important of the former coenzymes are collagen and elastin, whose fluorescence results from crosslinking between amino acids. Fluorophores involved in cellular metabolism include reduced nicotinamide adenine dinucleotide (NADH) and flavins. Other fluorophores include aromatic amino acids (e.g., tryptophan, tyrosine, phenylalanine, porphyrins, and lipopigments (e.g., ceroids and lipofuscin)) that are considered the end-products of lipid metabolism. Wagnières et al. and Monici investigated and reported the emission spectra of the main endogenous fluorophores of living cells. [45, 48].

**Fig. 4:**
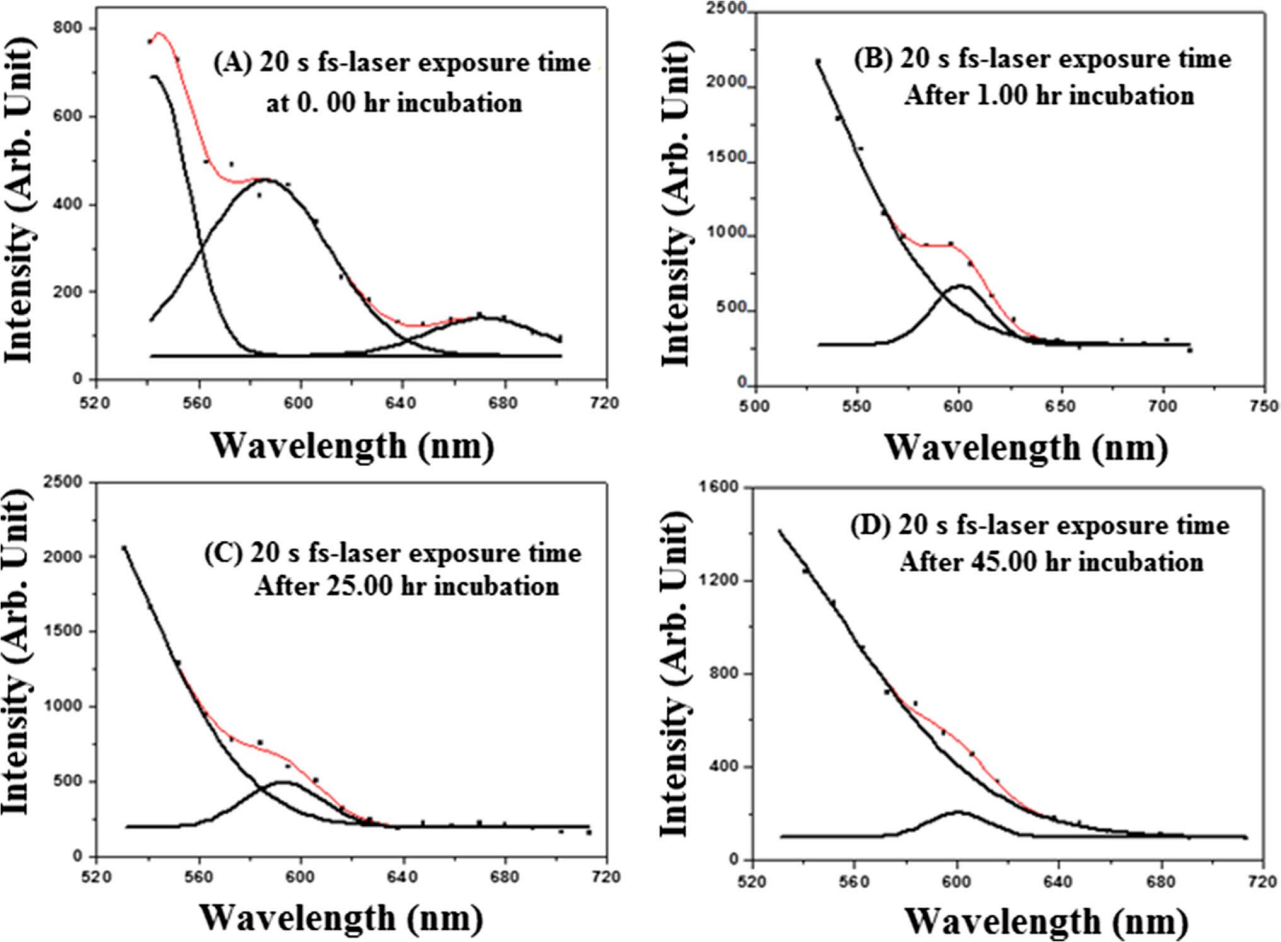
Autofluorescence spectra of fibroblast cells irradiated for 20 s with 90 fs laser beam at 800 nm wavelength and an average power of 320 mW. (The red line indicates the fitting curve of the experimental data.)

**Fig. 5:**
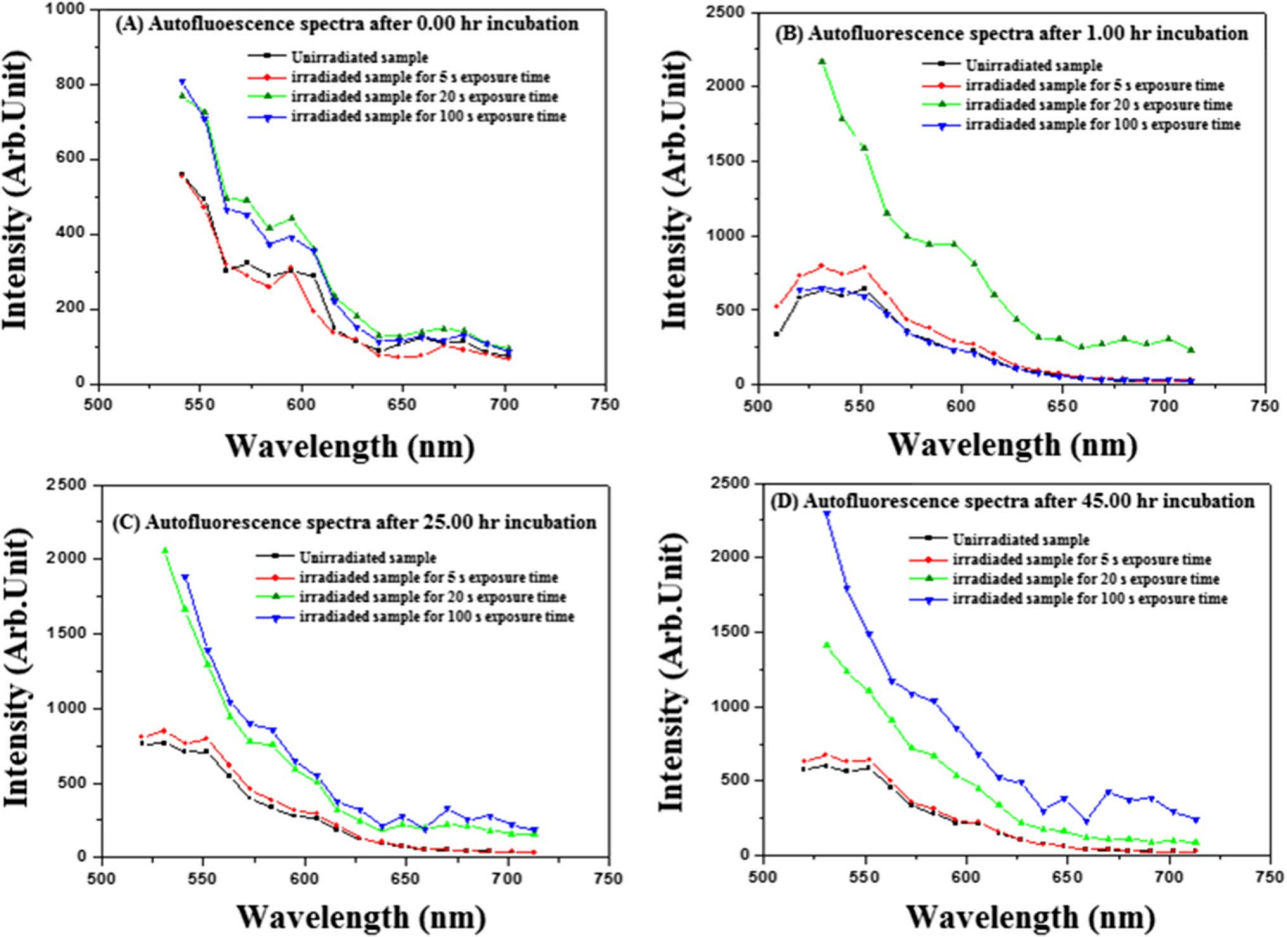
Autofluorescence spectra of the irradiated fibroblast cells wavelength and average power of 320 mW

In our experiment, the AF signals emitted in each case were recorded using a laser scanning microscope, as aforementioned. The signals were processed in every case, and the spectrum was analyzed and deconvoluted using the origin software. Figure 4s shows the AF spectrum of unirradiated fibroblasts at the beginning of the experiment. Two weak emission bands are located at 530–650 nm and 620–700 nm with central wavelengths of 590 and 670 nm, respectively. Figure 3A, B, C, and D show the deconvolution AF spectra of the fibroblasts irradiated with 90 fs laser beam for 5 s at a wavelength of 800 nm and average power of 320 mW (at the cell target) obtained after (A) 0:00-h, (B) 1:00-h, (C) 25:00-h, and (D) 45:00-h laser exposures. The signal was detected immediately after the laser irradiation. Figure 4A, B, C, and D show the deconvolution AF spectra of the fibroblasts irradiated with 90 fs laser beam for 20 s at a wavelength of 800 nm and average power of 320 mW (at the cell target) obtained after (A) 0:00-h, (B) 1:00-h, (C) 25:00-h, and (D) 45:00-h laser exposures. The signal was detected immediately after the laser irradiation. For comparison, Fig. 5 illustrates the registered AF spectra for all cases. Two and three emission bands are analyzed in this figure and are presented in Table 3. Noticeably, the emitted fluorescence intensity depends on the interacted total laser energy density.

**Table 3:** Emission bands, a maximum peak value (nm), and the peak intensity (arbitrary unit) of the unirradiated and irradiated (with fs laser) fibroblast cells on a glass plate after 0:00, 1:00. 25:00, and 45:00 h incubation time

Unirradiated cells	Incubation time			
	0.00 h	1.00 h	25.00 h	45.00 h
	Emission wavelength band (central W. L. nm, int. arbitrary unit)			
	620–720 (670, **180**) 540–670 (588, **300**)	540–675 (609, **400**) 500–625 (559, **650**) ND–625 (532, **720**)	500–600 (550, **700**) ND–650 (570, **200**)	ND–700 (570, **325**) 500–600 (550, **600**)
Irradiated cells/exposure time	Recorded time after laser irradiation			
	0.00 h	1.00 h	25.00 h	45.00 h
	Emission wavelength band (central W. L. nm, int. arbitrary unit)			
5 s	ND–670 (588**, 400**) ND–600 (535**, 850**)	ND–750 (540, **70**) 500–600 (550, **1700**)	ND–750 (520, **1900**) 500–600 (550, **2450**)	500–700 (600, **400**) ND–650 (520, **1400**)
20 s	600–700 (650, **140**) 500–650 (575, **450**)	500–700 (600, **35**) 500–600 (550, **200**)	550–700 (625, **400**) ND–700 (550, **850**)	ND–675 (590, **800** ND–650 (518, **2220**)
100 s	550–670 (600, **50**) ND–620 (535, **1100**)	ND–750 (571) ND–650 (527, **800)**	600–750 (677, **40**) 500–700 (590, **820**) 500–600 (550, **700**)	630–720 (645, **300**) ND–700 (560, **650**) 500–600 (550, **690**)

The number in Bold refers to the peak intensity

Figure 5A shows three emission bands at 620–720, 540–670, and 500–600 nm, with central wavelength peaks at 670, 588, and 542 nm, respectively. On the other hand, Fig. 3B shows two emission bands at 490–670 and 500–600 nm, with 588 and 535 nm central wavelengths. These emission bands are consistent with the corresponding results presented in reference [45].

The emission bands mentioned above correspond to the formation of coenzymes and chemical compounds, such as lipopigment, porphyrins, and flavins, within the fibroblasts’ cellular matrix. Figure 5A shows that the intensity of the emitted bands obtained at the beginning of the experiment is lower than the corresponding values obtained after 45.00-h incubation. The bands emitted from the cells exposed to a laser energy density of 452.9 J/cm^2^ were 2.7% higher than the corresponding value obtained from the unirradiated and irradiated cells with a radiant exposure of 22.6 J/cm^2^. In addition, these intensities are 1.6% higher than the corresponding values obtained from the cells irradiated at 90.6 J/cm^2^. Notably, the AF emission had a high intensity in the 20 and 100-s fluence irradiation. These values may be attributed to the increased generation rate of coenzymes from the surviving cells.

In medicine, fibroblasts produce and maintain the extracellular matrix, provide a structural framework for many tissues, and are crucial to wound healing. They have a branched cytoplasm surrounding an elliptical speckled nucleolus with one or two nuclei. Their abundant rough endoplasmic reticulum can identify them. Alternatively, the extracellular matrix may contain collagen, glycosaminoglycans, and glycoprotein. These cells secrete some chemical compounds to help them heal after exposure to UV, X-ray, and laser radiation [4, 48, 49]. The emission of these endogenous fluorophores is referred to as AF (or natural fluorescence). The most important compounds include proteins containing aromatic amino acids, the reduced form of pyridine nucleotides, flavins, and lipopigments. These compounds may act as coenzymes, such as flavoproteins, in which the flavin cofactor functions as an electron transfer intermediate in various biochemical reactions. Although the various flavin forms have characteristic absorption spectra visible and near UV, most flavoproteins’ physiological functions are light-independent. The catalytic repair cycle may be initiated by photoinduced electron transfer from the fully reduced flavin adenine dinucleotide cofactor to the substrate.

Alternatively, porphyrins and other closely related tetrapyrrolic pigments play significant roles in various biological processes, including electron transfer, oxygen transport, and catalytic substrate oxidation. A strong π dominates the electron absorption of porphyrins and metalloporphyrins to the π* ligand band [4]. The role of porphyrins in wound healing cannot be neglected in this context [50].

Table 3 shows the values of the AF bands obtained from the unirradiated and irradiated cells in the wavelength range of 500–750 nm. These values include the central wavelength and the peak intensity (in arbitrary units). We often observed three prominent emission band peaks. These peaks are at 530, 575, and 660 nm. Endogenous fluorophores and band identification are compared with the information provided in the reference [4, 49, 51]. It indicates the formation of peptides containing aromatic amino acids, NADH, flavins, and lipopigments [4].

The observed AF bands (Fig. 5) were compared with the data obtained by Croce et al. [4] (see Table 4). This fluorescence indicates that the emitted band’s intensity located at an average wavelength range of 600–700 nm corresponds to the porphyrin coenzyme. This band is weaker than the intensity of the emitted band situated in intermediate wavelength ranges of 500–600 and 500–700 nm, corresponding to flavins and lipopigment coenzymes, respectively. The AF signal intensity depends on the interacted laser energy density and the incubation time after laser irradiation. Following laser irradiation, the recovered cells may undergo structural changes and lose some of their molecular constituents, affecting their mitochondrial activity. The laser scanning microscope experiment used an argon laser operated at a wavelength of 488 nm to excite the fibroblasts. Consequently, we cannot detect signals from the generated coenzymes below this wavelength (488 nm).

**Table 4:** Endogenous fluorophores and the identification of the observed autofluorescence emitted

Endogenous fluorophores	Fluorophore constituents	Autofluorescence emission bands (nm)	
		This work	Data obtained from Ref. [4]*
NAD(P)H	Co-enzymes of key enzymes	ND–560 (480)	350–540 (460)
Flavins	Co-enzymes of key enzymes	500–600 ND–670	(480/540); 460–650 (520)
Fatty acids	Accumulated lipids	ND–650	470–480; 400–650 (470)
Vitamin A	Retinols and carotenoids	ND–675	490–510; 400–680 (480)
Protoporphyrin IX and porphyrin Derivatives	Protein prosthetic group	630–720	630–700
Lipofuscins/lipopigment	Proteins	500–700	480–700; 400–650; (460/530/590)
Porphyrins	Occur in protoplasm	600–700 (650)	600–700; (620/680)

*ND* not detected (see the explanation given in the text)

^*^The fibroblast is irradiated with UV light

It is expected that AF studies of cultured skin fibroblasts exposed to fs laser radiation can monitor cell activities. It provides direct information on how cells and tissues respond to external stimuli and cell-intrinsic features, such as cancer transformation.

## Conclusion

We cultured the human skin fibroblast cell line in this experiment on glass plates. The target cells are irradiated with 90 fs laser beam at a wavelength of 800 nm, with a repetition rate of 82 MHz and an average power of 320 mW. We conclude the following:

1. Exposed to ultra-short fs laser fluence, as used in this study, cells are partly killed or wounded, inducing an increase in residual viable fibroblasts proliferation and coenzyme formation.
2. A rise in the coenzyme formation, e.g., aromatic amino acids, NADH, flavins, porphyrins, and lipopigments, of the surviving cells, which correlated to the increase in auto-fluorescence, perhaps in response to high-intensity fs laser–induced injury
3. This study presents the potential of using fs laser radiation for photochemical cancer therapy, including post-traumatic cell regeneration and tumor cell regression.

### Supplementary Information

Below is the link to the electronic supplementary material.

Supplementary file1 (DOCX 337 KB)

## Data Availability

The authors confirm that all data are available with the manuscript.

## Declarations

The authors fully adopted all standard national and international ethical guidelines for a study involving a human primary cell line. The cell line used is commercially available. In vivo fibroblast extraction was not conducted in our experiment.

## References

[1] Ivanovski S, Haase Li HHR, Bartold PM (2001). Expression of bone-associated macromolecules by gingival and periodontal ligament fibroblasts. J Periodontal Res.

[2] Velnar T, Bailey T, Smrkolj V (2009). The wound healing process: an overview of the cellular and molecular mechanisms. J Int Med Res.

[3] Robson MC, Steed DL, Franz MG (2001). Wound healing: biologic features and approaches to maximize healing trajectories. Curr Probl Surg.

[4] Croce AC, Bottiroli G (2014) Autofluorescence spectroscopy, and imaging: a tool for biomedical research and diagnosis. Eur J Histochem 58(4). 10.4081/ejh.2014.2461 10.4081/ejh.2014.2461 PMC4289852 25578980

[5] Ma H, Yang J-P, Tan RK, Lee H-W, Han S-K (2018) Effect of low-level laser therapy on proliferation and collagen synthesis of human fibroblasts in vitro. J Wound Manag Res 14(1):1–6. 10.22467/jwmr.2018.00283

[6] Heitz J (2017). Femtosecond laser for inducing microstructures on Ti substrates for reducing cell adhesion. Appl Phys A.

[7] Lone SA, Muck M, Fosodeder P, Mardare CC, Florian C, Weth A, Krüger J, Steinwender C, Baumgartner W, Bonse J (2020). Impact of femtosecond laser treatment accompanied with anodization of titanium alloy on fibroblast cell growth. Phys Status Solidi.

[8] Hawkins D, Abrahamse H (2007). How long after laser irradiation should cellular responses be measured to determine the laser effect?. J Laser Appl.

[9] Khalid MZ (2016) Mechanism of Laser/light beam interaction at cellular and tissue level and study of the influential factors for the application of low-level laser therapy. Physics. med-ph. 10.48550/arXiv.1606.04800

[10] Kara N, Selamet H, Benkli YA, Beldüz M, Gökmenoğlu C, Kara C (2020). Laser therapy induces increased viability and proliferation in isolated fibroblast cells. Wounds.

[11] Robertson V, Ward A, Low J, Reed A (2006). Electrotherapy explained. Principles and practice.

[12] Kreisler M, Christoffers AB, Willershausen B, d'Hoedt B (2003). Effect of low-level GaAlAs laser irradiation on the proliferation rate of human periodontal ligament fibroblasts: an in vitro study. J Clin Periodontol.

[13] Schartinger VH, Galvan O, Riechelmann H, Dudas J (2012). Differential responses of fibroblasts, non-neoplastic epithelial cells, and oral carcinoma cells to low-level laser therapy. Support Care Cancer.

[14] Hawkins D, Abrahamse H (2006). Effect of multiple exposures of low-level laser therapy on the cellular responses of wounded human skin fibroblasts. Photomed Laser Surg.

[15] Gholami L (2020). Effects of 980nm diode laser irradiation on gingival fibroblasts and periodontal ligament stem cells. Acta Sci Microbiol.

[16] Pereira AN, Eduardo Cde P, Matson E, Marques MM (2002). Effect of low-power laser irradiation on cell growth and pro-collagen synthesis of cultured fibroblasts. Lasers Surg Med.

[17] Maldaner DR, Azzolin VF, Barbisan F, Mastela MH, Teixeira CF, Dihel A (2019). In vitro effect of low-level laser therapy on the proliferative, apoptosis modulation, and oxi-inflammatory markers of premature-senescent hydrogen peroxide-induced dermal fibroblasts. Lasers Med Sci.

[18] Crisan B, Soritau O, Baciut M, Campian R, Crisan L, Baciut G (2013). Influence of three laser wavelengths on human fibroblasts cell culture. Lasers Med Sci.

[19] Tuner J, Hode L (2007). Biostimulation in the laser therapy handbook: a guide for research scientists, doctors, dentists, veterinarians and other interested parties within the medical field.

[20] Kohli V, Acker JP, Elezzabi AY (2006) Permeabilization and cell surgery using femtosecond laser pulses: an emerging tool for cellular manipulation, Proc. SPIE 6084, Optical Interactions with Tissue and Cells XVII 608414. 10.1117/12.655618

[21] Joglekar A, Liu H, Spooner G (2003). A study of the deterministic character of optical damage by femtosecond laser pulses and applications to nanomachining. Appl Phys B.

[22] Chimmalgi A, Choi TY, Grigoropoulos CP, Komvopoulos K (2003). Femtosecond laser apertureless near-field nano-machining of metals assisted by scanning probe microscopy. Appl Phys Lett.

[23] Backus S, Durfee CG, Murnane MM, Kapteyn HC (1998). High power ultrafast lasers. Rev Sci Instrum.

[24] Keller U (2003). Recent developments in compact ultrafast lasers. Nature.

[25] Brabec T, Krausz F (2000). Intense few-cycle laser fields: frontiers of nonlinear optics. Rev Mod Phys.

[26] Steinmeyer G, Sutter DH, Gallmann L, Matuschek N, Keller U (1999). Frontiers in ultrashort pulse generation: pushing the limits in linear and nonlinear optics. Science.

[27] Thannickal VJ, Fanburg BL (2000). Reactive oxygen species in cell signaling. Am J Physiol Lung Cell Mol Physiol.

[28] Boyd RW (Ed.) (2003) Nonlinear optics, 2nd edn, Academic Press, Amsterdam. 10.1016/B978-0-12-121682-5.X5000-7/

[29] Krüger J, Kautek W (2004) Ultrashort pulse laser interaction with dielectrics and polymers. In: Lippert T (eds) Polymers and light. Advances in polymer science. Springer, Berlin, Heidelberg. 10.1007/b12683/

[30] Dausinger F, Lichtner F, Lubatschowski H (Eds) (2004) Femtosecond technology for technical and medical applications (Springer, Berlin). 10.1007/b96440/

[31] Gattass RR, Mazur E (2008). Femtosecond laser micromachining in transparent materials. Nat Photon.

[32] Bloembergen N (1997). A brief history of light breakdown. J Nonlinear Opt Phys.

[33] Invitrogen/Gibro Cell Culture Basics, Handbook. (n.d.) https://www.vanderbilt.edu/viibre/CellCultureBasicsEU.pdf/. Accessed June 2021

[34] Millennia™ IR Diode-pumped (1997) CW Infrared Laser, user manual; Part Number 0000–262A, Rev. A. November. https://www.spectraphysics.com/mam/celum/celum_assets/sp/resources/Millennia_IR_0000-262A_Rev_A.pdf/. Accessed June 2021

[35] Carl Zeiss operating Manual (2002) Carl Zeiss LSM 510 and LSM 510 META laser scanning microscopes, operating manual, Number: B 45–0008 e. https://ncxt.lbl.gov/files/lab/LSMmanual.pdf/. Accessed June 2021

[36] Lai SR, Phipps SM, Liu L, Andrews LG, Tollefsbol TO (2005). Epigenetic control of telomerase and modes of telomere maintenance in aging and abnormal systems. Front Biosci.

[37] Phipps SM, Berletch JB, Andrews LG (2007) Tollefsbol TO. Aging cell culture: methods and observations. Methods Mol Biol 371, Humana Press. 10.1007/978-1-59745-361-5_2 10.1007/978-1-59745-361-5_2 PMC2423218 17634570

[38] Cuerda-Galindo E, Díaz-Gil G, Palomar-Gallego MA, Linares-GarcíaValdecasas R (2015). Increased fibroblast proliferation and activity after applying intense pulsed light 800–1200 nm. Ann Anat.

[39] Mignon C, Uzunbajakava NE, Raafs B (2017). Photobiomodulation of human dermal fibroblasts in vitro: decisive role of cell culture conditions and treatment protocols on experimental outcome. Sci Rep.

[40] Sunkari VG, Aranovitch B, Portwood N, Nikoshkov A (2011). Effects of a low-intensity electromagnetic field on fibroblast migration and proliferation. Electromagn Biol Med.

[41] Górski R, Nowak-Terpiłowska A, Śledziński P, Baranowski M, Wosiński S (2021). Morphological and cytophysiological changes in selected lines of normal and cancer human cells under the influence of a radio-frequency electromagnetic field. Ann Agric Environ Med.

[42] Houreld N, Abrahamse H (2007). In vitro exposure of wounded diabetic fibroblast cells to a helium-neon laser at 5 and 16 J/cm2. Photomed Laser Surg.

[43] Pate K, Benghuzzi H, Tucci M, Puckett A, Cason Z (2003). Morphological evaluation of MRC fibroblasts after stimulation with static magnetic field and pulsating electromagnetic field. Biomed Sci Instrum.

[44] Hawkins D, Abrahamse H (2007). Time-dependent responses of wounded human skin fibroblasts following phototherapy. J Photochem Photobiol B: Biology.

[45] Wagnières GA, Star WM, Wilson BC (1998). In vivo fluorescence spectroscopy and imaging for oncological applications. Photochem Photobiol.

[46] Yang TS, Nguyen LTH, Hsiao YC, Pan LC, Chang CJ (2022). Biophotonic effects of low-level laser therapy at different wavelengths for potential wound healing. Photonics.

[47] Tam SY, Tam VCW, Ramkumar S, Khaw ML, Law HKW, Lee SWY (2020). Review on the cellular mechanisms of low-level laser therapy use in oncology. Front Oncol.

[48] Monici M (2005). Cell and tissue autofluorescence research and diagnostic applications. Biotechnol Annu Rev.

[49] Bundke U, Reimann B, Nillius B, Jaenicke R, Biagemer H (2010). Development of a Bioaerosol single particle detector (BIO IN) for the Fast Ice Nucleus CHamber FINCH, Atmos Meas. Tech.

[50] Vallejo M, Moura N, Gomes A, Joaquinito A, FaustinoM AA, Gonçalves I, Serra VV, Neves M (2021). The role of porphyrinoid photosensitizers for skin wound healing. Int J Mol Sci.

[51] Seeger S (2004) Ultrasensitive fluorescence detection at surfaces: instrument development, surface chemistry, and applications in life science and medicine, Editors: Giuseppe Palumbo, Riccardo Pratesi, Lasers and current optical techniques in biology, Chapter 20, Royal Society of Chemistry. https://bd.b-ok.africa/book/2167934/01b577/. Accessed Nov 2022

