# Supplementary material for: Effect of femtosecond laser interaction with human fibroblasts: a preliminary study
Source: Lasers Med Sci. 2023 Mar 3;38(1):83. doi: 10.1007/s10103-023-03740-2 (PMC9984333; doi:10.1007/s10103-023-03740-2)
Supplement: Supplementary file 1 — Supplementary file1 (DOCX 337 KB) [file 10103_2023_3740_MOESM1_ESM.docx]

**
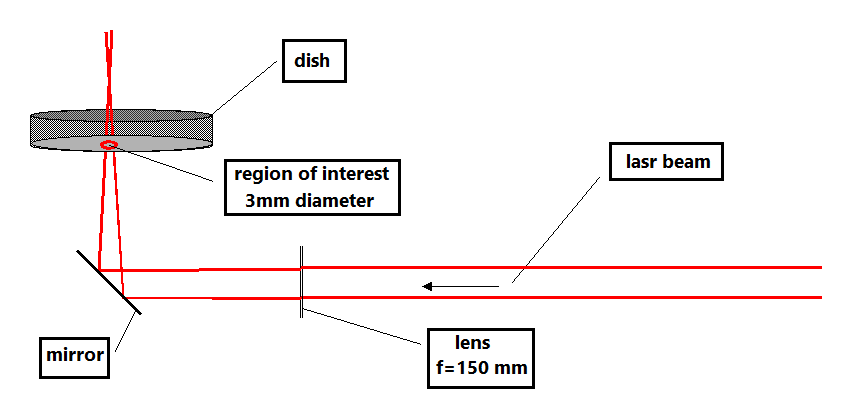
**

**Fig. 1s: schematic optical setup of the laser irradiation system**


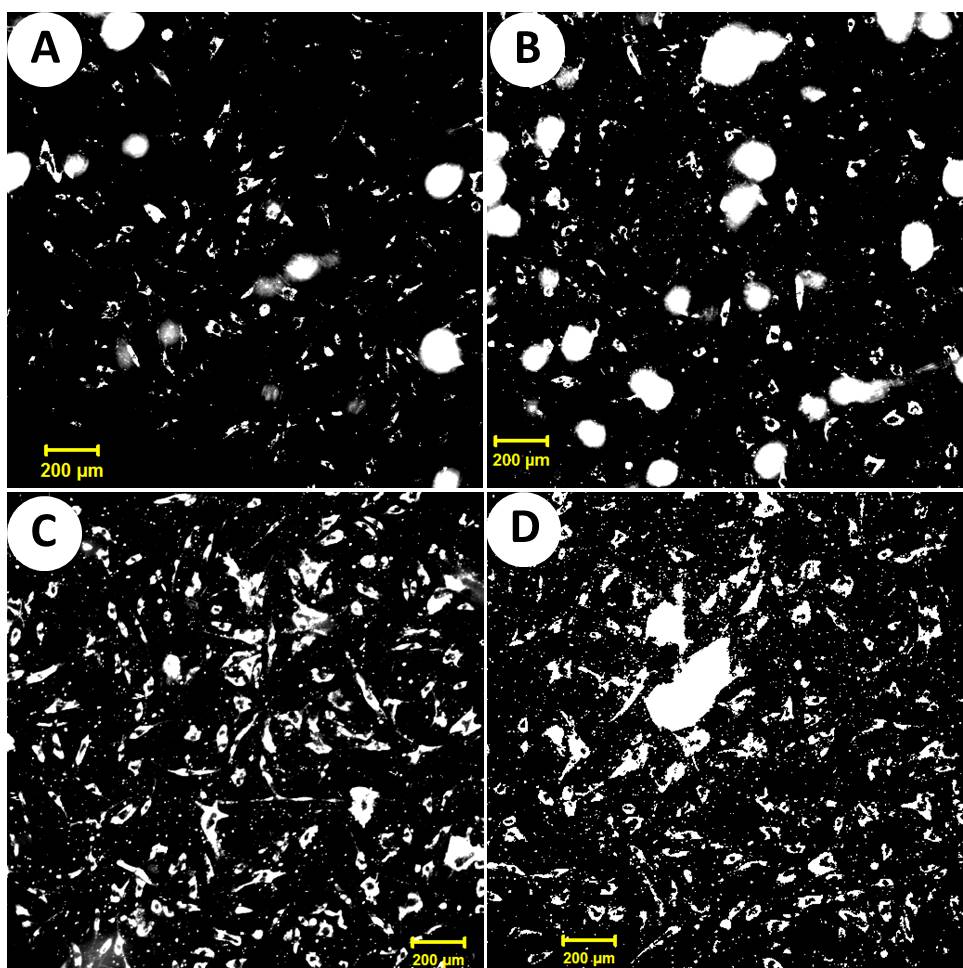


**Fig. 2s: Cell count images of the unirradiated fibroblasts cultured on a glass plate after A) 0.00 hr, B) 1:00 hr, C) 25:00 hr, and D) 45:00 hr incubation time. It shows the average count of the cells at different times of incubation.**

**
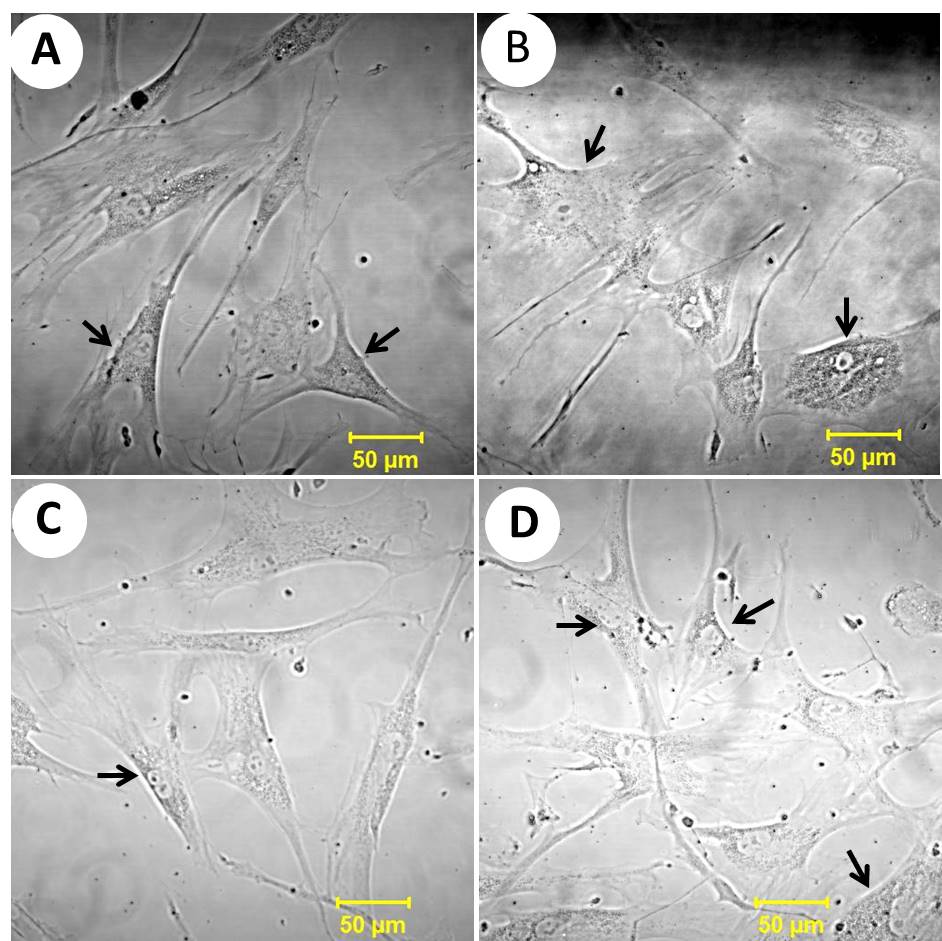
**

**Fig. 3s: Normal morphology of the unirradiated fibroblasts cultured on a glass plate after A) 0.00 hr, B) 1:00 hr, C) 25:00 hr, and D) 45:00 hr incubation time. It shows the average count of the cells at different times of incubation.**


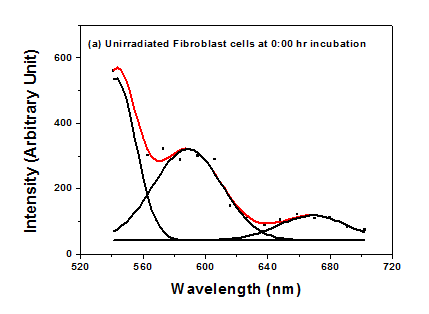


**Fig. 4s: Autofluorescence spectrum of unirradiated fibroblast** **cells**

**(The red line is the fitting curve of the experimental data)**
